# Neuropilin-1 as a Potential Biomarker of Prognosis and Invasive-Related Parameters in Liver and Colorectal Cancer: A Systematic Review and Meta-Analysis of Human Studies

**DOI:** 10.3390/cancers14143455

**Published:** 2022-07-15

**Authors:** Paula Fernández-Palanca, Tania Payo-Serafín, Flavia Fondevila, Carolina Méndez-Blanco, Beatriz San-Miguel, Marta R. Romero, María J. Tuñón, Jose J. G. Marin, Javier González-Gallego, José L. Mauriz

**Affiliations:** 1 Institute of Biomedicine (IBIOMED), Campus of Vegazana s/n, University of León, 24071 León, Spain; pferp@unileon.es (P.F.-P.); tpays@unileon.es (T.P.-S.); ffonp@unileon.es (F.F.); cmenb@unileon.es (C.M.-B.); bsanv@unileon.es (B.S.-M.); mjtung@unileon.es (M.J.T.); jgonga@unileon.es (J.G.-G.); 2 Centro de Investigación Biomédica en Red de Enfermedades Hepáticas y Digestivas (CIBERehd), Instituto de Salud Carlos III, Av. de Monforte de Lemos 5, 28029 Madrid, Spain; marta.rodriguez@usal.es (M.R.R.); jjgmarin@usal.es (J.J.G.M.); 3 Experimental Hepatology and Drug Targeting (HEVEPHARM), Salamanca Biomedical Research Institute (IBSAL), University of Salamanca, 37007 Salamanca, Spain

**Keywords:** clinicopathological features, colorectal cancer, hepatocellular carcinoma, invasion, liver cancer, neuropilin-1, prognosis, therapeutic target

## Abstract

**Simple Summary:**

Neuropilin-1 (NRP1) is a transmembrane protein which has had recently increased interest from cancer researchers. Liver cancer and colorectal cancer (CRC) are two of the most frequent and deadly tumors worldwide. Here, we assessed the prognostic, diagnostic and clinicopathological value of NRP1 in liver cancer and CRC patients by systematic searches in PubMed, Scopus, Web of Science, Embase and Cochrane Library and a meta-analysis. Results obtained showed that NRP1 overexpression was significantly correlated with lower survival in liver cancer patients and with tumor development in hepatocarcinoma patients, and high levels of NRP1 were strongly correlated with an increased risk of vascular invasion in liver cancer and metastasis in CRC and liver tumors. Therefore, these findings could establish novel interest of NRP1 as a useful biomarker for patient prognosis as well as for invasive-related characteristics in patients with liver cancer or CRC.

**Abstract:**

Neuropilin-1 (NRP1) is a transmembrane protein involved in numerous cellular functions which has had increasing interest from cancer researchers. Liver cancer and colorectal cancer (CRC) are two of the most frequent and deadly tumors with a complex pharmacological framework. Here, we assessed the prognostic, diagnostic and clinicopathological value of NRP1 in liver cancer and CRC patients. We searched PubMed, Scopus, Web of Science, Embase and Cochrane Library databases for articles evaluating the NRP1 correlation with survival parameters, tumor development or clinicopathological features. Hazard ratios and odds ratios with 95% confidence intervals were extracted or estimated by Parmar method and pooled to evaluate the overall effect size with STATA 16 software. Heterogeneity was analyzed by chi-square-based Q test and I^2^ statistic, along with meta-regression and subgroup analysis, and publication bias was assessed by funnel plot asymmetry and Egger’s test. The study protocol was registered in PROSPERO (CRD42022307062). NRP1 overexpression was significantly correlated with lower survival in liver cancer patients and with tumor development in hepatocarcinoma patients, and was strongly correlated with an increased risk of vascular invasion in liver cancer and metastasis in CRC and liver tumors. These results support the role of NRP1 as a potential and useful biomarker in both types of cancer.

## 1. Introduction

Liver cancer and colorectal cancer (CRC) are two of the main leading causes of cancer death worldwide [1,2] and two of the most frequent and deadly gastrointestinal tumors, standing as the third and sixth in terms of incidence and the second and third in terms of mortality, respectively [3]. These solid tumors are characterized by complex pathophysiology and molecular heterogeneity [2,4], which explains the different cancer subtypes that constitute both liver and CRC cancers. Primary liver tumors mainly comprise hepatocellular carcinoma (HCC), which accounts for 75–85% of cases, and cholangiocarcinoma (CCA), representing 10–15% of cases [2,3]. On the other hand, CRC is usually considered as a combination of colon, rectum and anus tumors, and colon cancer is also differentiated into right-sided (proximal) and left-sided (distal) [3,4]. Both tumor types, liver cancer and CRC, share asymptomatic early stages and are mostly diagnosed at advanced phases when curative treatments are not available and palliative chemotherapy is selected [4,5,6]. Moreover, due to the highly vascularized nature of both solid tumors, targeted therapies against key pathways in angiogenesis and metastasis, such as vascular endothelial growth factor (VEGF) and its receptors (VEGFR), constitute the standards of treatment for advanced liver and colorectal tumors currently [4,7,8]. However, despite the effectiveness shown by current treatments, mortality rate is still high and predicted to continue increasing in the future [3]. Likewise, even when early diagnosis and selection of curative therapeutic options are possible, the recurrence rates in liver and colorectal tumors remain elevated, being 50–70% and 50%, respectively [2,5,9].

Neuropilin-1 (NRP1) is a transmembrane glycoprotein, firstly described in the nervous system as an axon guidance molecule, but with a broad variety of functions, including immune response, cell survival, angiogenesis, invasion and migration [10,11]. This protein is mainly located in the cell membrane, where it interacts with numerous proteins; however, there are soluble variants of NRP1 (sNRP1) that also exert modulatory actions on cell signaling [11]. The role of NRP1 in cancer has become of recent interest due to its ability to act as a co-receptor of important receptor tyrosine kinases (RTKs), such as VEGFR, hepatocyte growth factor receptor (MET), platelet-derived growth factor receptor (PDGFR) and transforming growth factor-beta receptor (TGF-βR), among others [10,11,12]. It has been described that NRP1 could drive not only tumor progression [10] but also tumor pathogenesis [13]. This has been associated with the NRP1-derived induction of angiogenesis that leads to oxygen and nutrients supply to the cancer cells [13]. Moreover, NRP1 overexpresses in several tumor types, including HCC, CCA and CRC, being related to a malignant phenotype and promotion of cell migration [14,15,16]. Nonetheless, despite the biological and clinical evidence supporting the critical role of NRP1 in cancer, there is a lack of studies that assess the potential association between NRP1 overexpression and clinical outcomes in cancer [17]. Only one meta-analysis has been conducted assessing the role of this receptor in gastric cancer patients [17].

Considering this and the high incidence and mortality rates of liver cancer and CRC that place them as two of the most common and deadly tumors of the gastrointestinal tract [3], we decided to select these tumor types for conducting the present meta-analysis. Therefore, in this study, we performed the first systematic review with meta-analysis of all available studies to evaluate the potential relation of increased levels of NRP1 with clinical prognosis and different tumor-associated clinicopathological features in human patients diagnosed with liver cancer or CRC.

## 2. Materials and Methods

### 2.1. Study Objectives

The purpose of this investigation was to evaluate the prognostic and diagnostic capacity of NRP1 expression in patients diagnosed with liver cancer or CRC, by analyzing the association of NRP1 expression with survival parameters, as well as several clinicopathological characteristics.

This systematic review with meta-analysis was conducted following the Preferred Reporting Items for Systematic Reviews and Meta-Analyses (PRISMA) guidelines (Tables S1 and S2) [18]. Additionally, the study protocol was registered in the International Prospective Register for Systematic Reviews (PROSPERO) and was ascribed the CRD42022307062 registration code.

### 2.2. Literature Search Strategy

An extensive literature search was performed in PubMed, Scopus, Web of Science (WOS), Excerpta Medica Database (Embase) and the Cochrane Library databases up to and including 31st May 2022. Studies eligible for this meta-analysis were identified employing the following search strategy: (“nrp1” OR “nrp-1” OR “nrp 1” OR “neuropilin 1” OR “neuropilin-1” OR “CD304” OR “VEGF165R”) AND (“hepatocellular carcinoma” OR “hepatocarcinoma” OR “HCC” OR “liver tumor” OR “hepatic tumor” OR “liver cancer” OR “hepatic cancer” OR “cholangiocarcinoma” OR “CCA” OR “hepatoma” OR “hepatoblastoma” OR “angiosarcoma” OR “colorectal cancer” OR “colon cancer” OR “CRC” OR “mCRC” OR “colon adenocarcinoma”) (Table S3).

### 2.3. Inclusion and Exclusion Criteria

Articles that met the following criteria were included in this analysis: (1) patients diagnosed with liver cancer or CRC; (2) evaluation of NRP1 expression either in tumor tissue or tumor-derived sources; (3) association of NRP1 levels with survival parameters or clinicopathological features with data reported or that can be extracted; (4) full text in English.

Articles complying with the following criteria were excluded: (1) studies conducted exclusively on cell or animal models; (2) reviews, book chapters, conference papers and similar; (3) articles without required data or in which data cannot be estimated; (4) full text in English not available.

### 2.4. Data Extraction and Quality Assessment

The screening of studies, data extraction and quality assessment of included articles was independently carried out by two authors. All discrepancies were solved by discussion and final consensus.

The Newcastle-Ottawa scale (NOS) was used to evaluate and determine the quality of selected studies, scoring from 0 to 9 [19]. Articles with NOS scores ≥ 5 were considered high quality and were included in the quantitative synthesis, whereas articles scoring < 5 were considered low quality and were excluded.

Baseline characteristics of each selected study were extracted and compiled in Table 1. Antibodies and staining procedures used in the included articles analyzing NRP1 expression by immunohistochemistry (IHC) were also recorded in Table S4.

### 2.5. Statistical Analysis

The statistical software STATA version 16 (Stata Corporation, College Station, TX, USA) was employed to evaluate the prognostic and diagnostic value of NRP1 expression in liver cancer and CRC in two steps, as indicated below.

We pooled the overall survival (OS), recurrence-free survival (RFS) and progression-free survival (PFS) by hazard ratio (HR) and 95% confidence interval (CI) to assess the association between NRP1 expression and cancer prognosis. OS, RFS and PFS were determined from the time of the intervention until the last follow-up date, recurrence or decease of the patient. Parmar method [20] was used to estimate these data when no information was directly reported in the primary study. HR and 95% CI were combined throughout the studies.

The correlation between NRP1 and tumor presence and clinicopathological parameters was determined by odds ratio (OR) with 95% CI. Combined HR > 1 and OR > 1 indicated a higher risk of poor prognosis and higher incidence of the several features analyzed, respectively, associated with elevated levels of NRP1. These correlations were considered significant when *p*-value < 0.05.

We assessed heterogeneity by chi-square-based Q test as well as I^2^ statistic, which ranged from 0% (no heterogeneity) to 100% (maximal heterogeneity), indicating inconsistency across studies. Heterogeneity was considered significant when Q test *p*-value was <0.10 and/or I^2^ ≥ 50%, in which cases the Restricted Maximum Likelihood (REML) procedure was employed as the random-effect model. Otherwise, the fixed-effects model with the Inverse Variance (IV) method was used.

We assessed the possible sources of variability between studies by performing meta-regression, as well as subgroup analyses based on tumor type, sample size, NOS score and follow-up.

Publication bias was determined by analyzing funnel plot asymmetry along with Egger’s test, which was significant when asymmetry was found and Egger’s *p*-value was <0.05. When publication bias existed, the trim-and-fill method was employed to estimate a corrected effect size adjustment in order to determine whether the pooled results were considerably affected by publication bias.

## 3. Results

### 3.1. Study Selection and Study Characteristics

**Figure 1:**
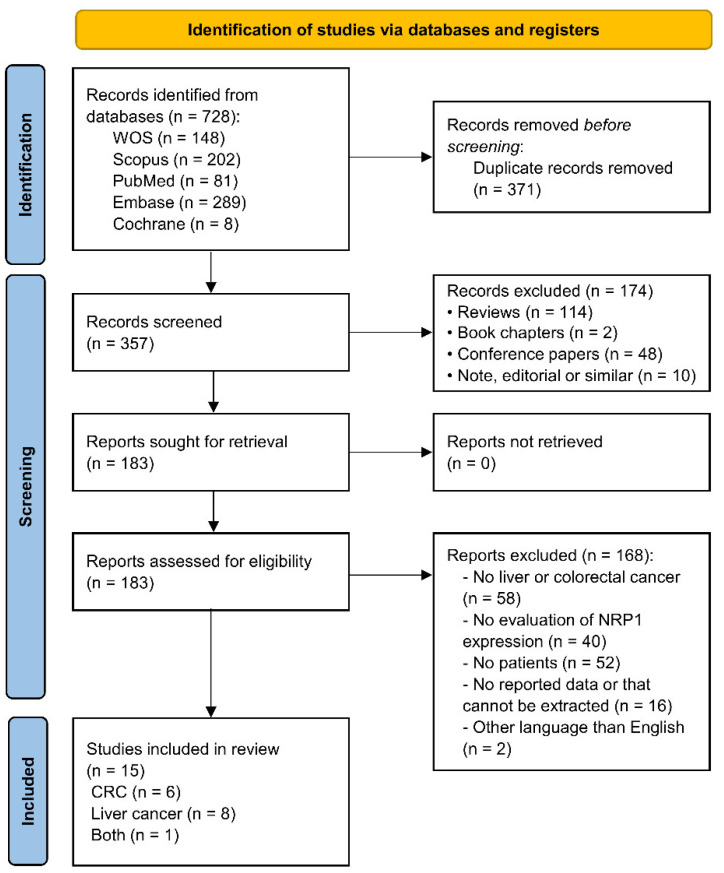
PRISMA flow diagram of study selection. CRC, colorectal carcinoma; Embase, Excerpta Medica Database; NRP1, neuropilin-1; WOS, Web of Science.

An electronic search was performed in five major databases, identifying a total of 728 articles. After duplicate removal (*n* = 371), screening by title and abstract (*n* = 174) and full-text screening (*n* = 168), 15 eligible articles were finally included for data extraction and quantitative analysis [14,16,21,22,23,24,25,26,27,28,29,30,31,32,33]. Out of the 15 studies, six were conducted in CRC patients, eight in liver cancer patients (specifically six in HCC patients and two in CCA patients), and one was a pan-cancer study that included all the tumor types (Figure 1). All studies complied with the quality threshold stablished based on NOS score (Table 1).

**Table 1:** Baseline characteristics of included studies.

Study	Publication Year	Tumor Type	Tumor Sample Size (M/F)	Non-Tumor Sample Size	Intervention	Pre- or Post-Surgery Treatment	Study Quality (NOS Score)	Measurement of NRP1 Expression	Cut-Off Value for “High” NRP1 Expression	Tumor Samples with “High” NRP1 (%)	Non-Tumor Samples with “High” NRP1 (%)	Parameter Analyzed	HR
Deng et al. [29]	2021	HCC, CCA, CRC	845 (NR)	NA	NR	NR	5/9	Tissue levels-RNA-seq	>median	25.09 (25.20,25.00, 25.00)	NA	OS	Reported
Li et al. [32]	2021	HCC	239 (215/24)	16	Curative hepatic resection	No	7/9	Tissue levels-IHC	>1 ^a^	57.74	37.50	OS CF Pathogenesis	Estimated
Liu et al. [33]	2021	CRC (COAD)	279 (154/125)	NA	NR	NR	6/9	Tissue levels-RNA-seq	NR	25.09	NA	OS	Estimated
Bianconi et al. [28]	2020	CRC	74 (54/20)	NA	Surgery	Yes ^b^	6/9	Tissue levels-IHC	>1 ^c^	NR	NA	OS, PFS	Reported
Giannelli et al. [30]	2020	HCC	149 (127/22)	NA	No	Yes ^d^	6/9	Serum levels	>median	NR	NA	OS	Reported
Wu et al. [23]	2020	CCA (ICC)	291 (174/117)	55	Surgery	No	7/9	Tissue levels-IHC and qRT-PCR ^e^	Strong ^f^	64.60	NR	OS, RFS CF	Estimated
Zhu et al. [16]	2020	CCA	39 (24/15)	39	NR	No	7/9	Tissue levels-IHC and qRT-PCR ^g^	>mean	46.15	NR	CF	NR
Lin et al. [14]	2018	HCC	40 (28/12)	30	Surgery	No	6/9	Tissue levels-IHC	NR	72.50	0.00	Pathogenesis	NR
Benson et al. [26]	2016	CRC	162 (NR)	NA	No	Yes ^h^	5/9	Serum levels	≥median	50.62	NA	PFS	Estimated
Zhang et al. [25]	2016	HCC	105 (77/28)	105	Surgery	No	7/9	Tissue levels-IHC	>3 ^i^	53.33	20.95	OS, RFS Pathogenesis CF	Estimated
Spencer et al. [22]	2013	CRC	583 (NR)	NA	No	Yes ^j^	5/9	Serum levels	>median	48.71	NA	OS, PFS	Reported
Yaqoob et al. [24]	2012	HCC	93 (NR)	NA	Surgery	No	5/9	Tissue levels-PCR array	>75% of expression	63.44	NA	OS	Estimated
Berge et al. [27]	2011	HCC	308 (NR)	31	Surgery	No	6/9	Tissue levels-IHC	≥1 ^k^	50.65	0.00	Pathogenesis	NR
Kamiya et al. [31]	2006	CRC	54 (NR)	54	Surgery	No	6/9	Tissue levels-qRT-PCR	≥0.5 ^l^	37.04 for survival-22.22 for CF	62.96	OS CF Pathogenesis	Estimated
Ochiumi et al. [21]	2006	CRC	103 (NR) for survival-146 (91/55) for CF	NA	Surgery	Yes ^m^	6/9	Tissue levels-IHC	SIS+MVS > 3.64 ^n^	60.19 for survival-65.07 for CF	NA	OS CF	Estimated

CCA, cholangiocarcinoma; CF, clinicopathological features; COAD, colon adenocarcinoma; CRC, colorectal cancer; F, female; HCC, hepatocellular carcinoma; HR, hazard ratio; ICC, intrahepatic cholangiocarcinoma; IHC, immunohistochemistry; M, male; MVS, mean value score; NA, not applicable; NR, not reported; NRP1, neuropilin-1; PCR, polymerase chain reaction; qRT-PCR, reverse transcription-quantitative polymerase chain reaction; SIS, staining intensity scale. ^a^ Semi-quantitative analysis of NRP1 expression was performed based on the density of cells staining as follows: (0) <5%; (1) 6–35%; (2) 36–70%; (3) >70%. Specimens with scores of 0 and 1 are regarded as low expression of NRP1 (NRP1Low), while specimens with scores of 2 and 3 are classified as high expression of NRP1 (NRP1High). ^b^ Arm A: XELIRI plus bevacizumab followed by XELOX plus bevacizumab; Arm B: XELOX plus bevacizumab followed by XELIRI plus bevacizumab. ^c^ Staining was scored by adding the distribution score (0 = no staining; 1+ = staining of <33% of cells; 2+ = between 33% and 66% of cells; and 3+ = staining of >66% of cells) to the intensity score (0 = no staining; 1+ = weak; 2+ = moderate; 3 = strong). ^d^ Patients who had received sorafenib and had progressed or were ineligible for sorafenib were included. After surgery, patients were treated with galunisertib. ^e^ IHC for survival and clinicopathological analysis, and qRT-PCR for normal and tumoral tissue comparison. ^f^ The intensity was scored as follows: 0, negative; 1, weak; 2, moderate; and 3, strong. The frequency of positive cells was defined as follows: 0, less than 5%; 1, 5–25%; 2, 26–50%; 3, 51–75%; and 4, greater than 75%. ^g^ IHC for clinicopathological analysis, and qRT-PCR for normal and tumoral tissue comparison. ^h^ Patients were randomized 2:1 to tivozanib/mFOLFOX6 (Arm A) or bevacizumab/mFOLFOX6 (Arm B). ^i^ Score obtained from multiplying staining intensity and percent of positive cells. Staining was scored as follows: absent staining (negative, 0), weak staining (1), moderate staining (2), and strong staining (3). The percent of positive cell was also scored following 4 categories, in which 1 was given for 0–10%, 2 for 11–50%, 3 for 51–80%, and 4 for 81–100%. ^j^ FOLFOX/XELOX plus cediranib or placebo. ^k^ Based on a four-tiered intensity scoring system as follows: 0, no staining; 1, weak staining; 2, moderate staining; 3, strong staining. ^l^ High expression of NRP1 when levels were 0.5% higher than in extraneoplasic tissue. ^m^ All patients with liver metastasis were treated with adjuvant chemotherapy after surgical resection. ^n^ SIS defined as staining intensity scale (from 0 to 3): with 0 representing no detectable staining, 1 representing faint staining, equivalent to that of apical or lateral colonic epithelial cells or mononuclear cells in adjacent normal tissue, 2 representing moderate staining, and 3 representing strong staining; and MVS defined as mean value score: obtained from each of the six fields examined per case graded on a scale of 0–3, with 0 representing 0–10%, 1 representing 10–30%, 2 representing 30–60%, and 3 representing 60–100%.

### 3.2. Overall Survival

**Figure 2:**
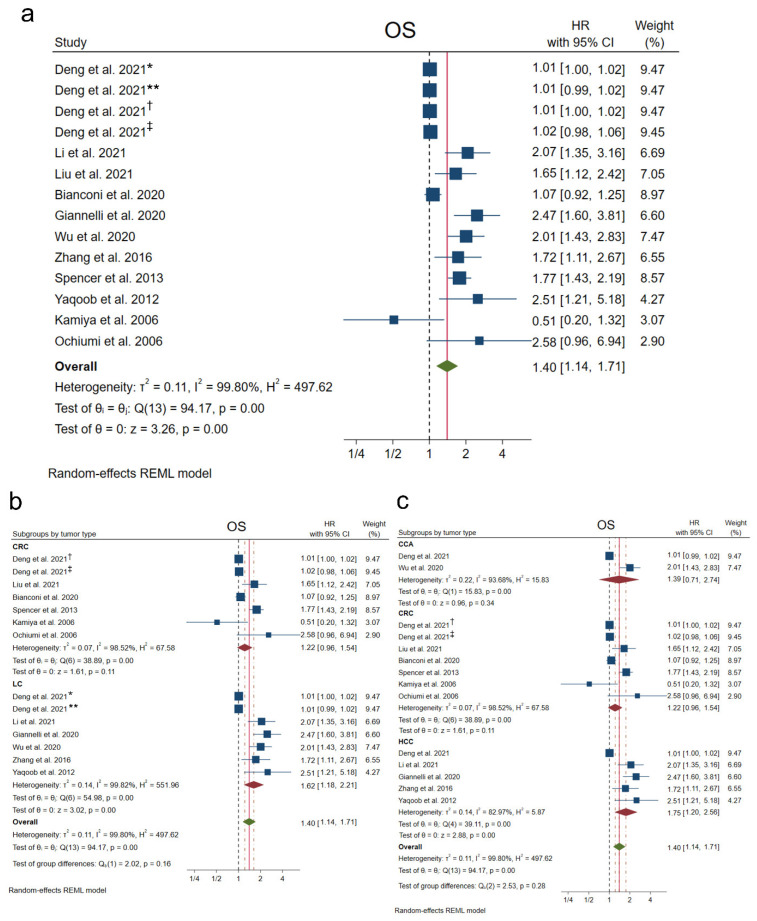
Forest plots showing HR for the association between NRP1 overexpression and OS among (**a**) all included studies, [21,22,23,24,25,28,29,30,31,32,33] (**b**) separately CRC and liver cancer studies, and (**c**) separately for CCA, CRC and HCC studies. CCA, cholangiocarcinoma; CI, confidence interval; CRC, colorectal cancer; HCC, hepatocellular carcinoma; HR, hazard ratio; LC, liver cancer; OS, overall survival; REML, Restricted Maximum Likelihood. * HCC patients, ** CCA patients, ^†^ colon adenocarcinoma patients, ^‡^ rectum adenocarcinoma patients.

The clinical association of NRP1 with OS was analyzed in 11 studies, the only ones that provided survival-associated data, finding that high expression of NRP1 was significantly correlated with a lower survival probability of liver cancer and CRC patients (HR 1.40, 95% CI 1.14–1.71, *p <* 0.001). However, heterogeneity among studies was also found to be significantly high (I^2^ = 99.80%, Q-test *p* < 0.001) (Figure 2a).

When analyzing survival probability differentially in liver cancer and CRC, a significant association was only found between NRP1 overexpression and lower survival in liver cancer patients (HR 1.62, 95% CI 1.18–2.21, *p <* 0.001), not observing this association in CRC patients (HR 1.22, 95% CI 0.96–1.54, *p =* 0.11) (Figure 2b). Remarkably, within liver cancer, this significant correlation was exclusive to patients with HCC (HR 1.75, 95% CI 1.20–2.56, *p <* 0.001), and was not significantly correlated in CCA patients (HR 1.39, 95% CI 0.71–2.74, *p =* 0.34) (Figure 2c). In these cases, marked heterogeneity was also observed for all tumor types (Figure 2b,c).

### 3.3. Recurrence-Free Survival and Progression-Free Survival

The prognosis-associated parameters RFS and PFS were also evaluated in five of the included articles.

**Figure 3:**
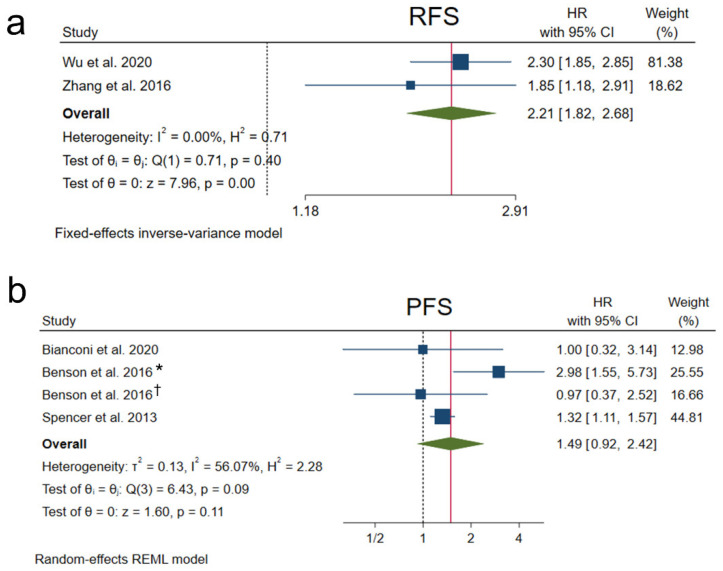
Forest plots showing HR for studies assessing the association of NRP1 overexpression and (**a**) RFS or (**b**) PFS. CI, confidence interval; HR, hazard ratio; PFS, progression-free survival; REML, Restricted Maximum Likelihood; RFS, recurrence-free survival. [22,23,25,26,28] * Tivozanib/mFOLFOX6 group, ^†^ bevacizumab/mFOLFOX6 group.

Two studies performed on liver cancer provided data for RFS analysis [23,25], which showed a significant correlation of high NRP1 levels with lower RFS (HR 2.21, 95% CI 1.82–2.68, *p <* 0.001), not finding significant heterogeneity (Figure 3a).

Regarding PFS, three studies conducted only in CRC patients assessed PFS correlation with differential expression of NRP1 [22,26,28]. However, a significant association was not observed between overexpression of NRP1 and lower PFS (HR 1.49, 95% CI 0.92–2.42, *p =* 0.11) and, contrarily, substantial heterogeneity among studies was observed (I^2^ = 56.07%, Q-test *p =* 0.09) (Figure 3b).

### 3.4. Tumor Pathogenesis

**Figure 4:**
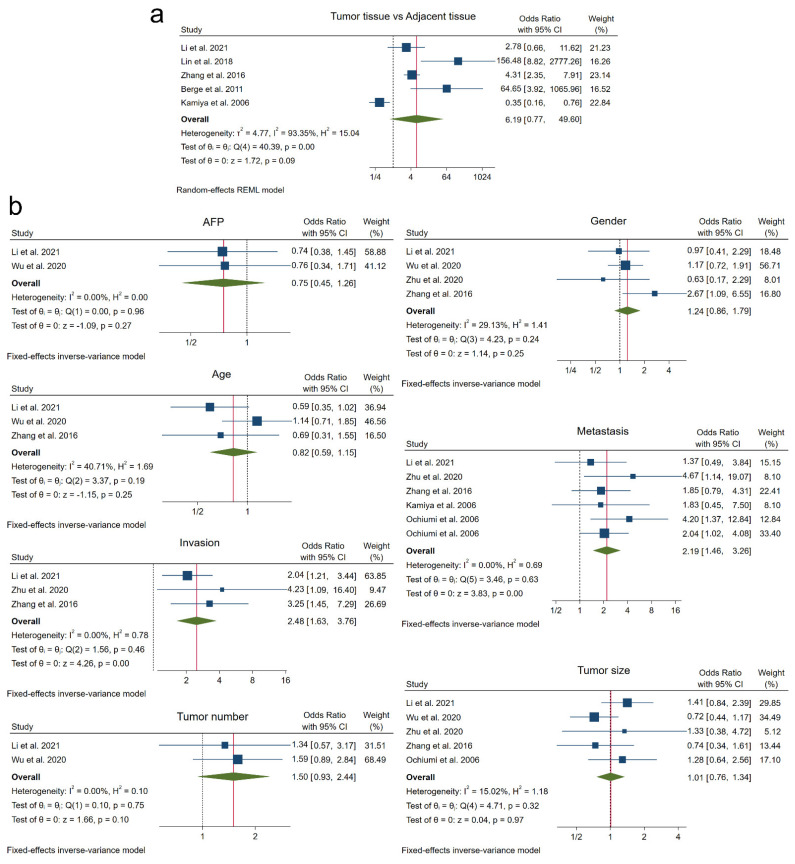
Forest plots showing OR for the evaluation of the relationship between NRP1 overexpression and (**a**) tumor pathogenesis, and (**b**) different clinicopathological features in cancer patients. AFP, alpha-fetoprotein; CI, confidence interval; OR, odds ratio; REML, Restricted Maximum Likelihood [14,16,21,25,27,31,32].

Among included studies, five different ones performed with HCC patients evaluated the potential association of high NRP1 levels with tumor development by comparing tumor tissue to non-tumor adjacent tissue [14,25,27,31,32]. Results from the meta-analysis performed exhibited that this correlation is not statistically significant (OR 6.19, 95% CI 0.77–49.60, *p =* 0.09), which could be because of the high heterogeneity obtained among studies (I^2^ = 93.35%, Q-test *p <* 0.001) (Figure 4a).

### 3.5. Tumor-Associated Clinicopathological Features

Due to the clinical relevance in cancer patients, we pooled available data for different clinicopathological characteristics, including alpha-fetoprotein (AFP) levels, patient age, patient gender, vascular invasion, metastasis, tumor number and tumor size (Figure 4b). Overall effect size showed that NRP1 overexpression was not correlated with AFP levels higher than 20 ng/mL (AFP, OR 0.75, 95% CI 0.45–1.26, *p =* 0.27), patients older than 50 years (age, OR 0.82, 95% CI 0.59–1.15, *p =* 0.25), male population (gender, OR 1.24, 95% CI 0.86–1.79, *p =* 0.25), presence of more than one nodule (tumor number, OR 1.50, 95% CI 0.93–2.44, *p =* 0.10) and tumor size larger than 5 cm (tumor size, OR 1.01, 95% CI 0.76–1.34, *p =* 0.97). Nevertheless, a significant correlation was observed between high expression of NRP1 and the presence of vascular invasion (invasion, OR 2.48, 95% CI 1.63–3.76, *p <* 0.001) in liver cancer studies and metastasis (OR 2.19, 95% CI 1.46–3.26, *p <* 0.001) in both tumor types (Figure 4b).

Heterogeneity between studies was also determined for the clinicopathological features analyzed, finding in all cases an assumable heterogeneity (I^2^ < 50% and Q-test *p* > 0.10) (Figure 4b).

### 3.6. Meta-Regression

To assess and examine the potential sources for the heterogeneity observed in the parameters OS, PFS and tumor pathogenesis, meta-regression was performed employing sample size, follow-up time or NOS score as moderators (Table 2) (Figure S1).

As observed in Table 2, neither sample size, follow-up or NOS score could explain the heterogeneity found in the meta-analysis of OS and NRP1 overexpression, since residual heterogeneity was still high after meta-regression (I^2^ = 99.68%, Q-test *p <* 0.001; I^2^ = 99.74%, Q-test *p <* 0.001; I^2^ = 99.68%, Q-test *p <* 0.001; respectively). However, the NOS score was found to be the covariate that could explain most heterogeneity, at least 42.46% of the initially observed heterogeneity (R^2^ = 42.46%), and also showed a stronger correlation between observed studies and predicted values (Figure S1a). When separately analyzing liver cancer and CRC samples regarding OS, different results were obtained after meta-regression. In CRC studies, sample size explained 59.33% of heterogeneity, while 61.08% was explained by NOS quality score in studies conducted in liver cancer patients; nonetheless, residual heterogeneity was still substantial in both cases after meta-regression (Table 2) and graphical representations of meta-regression showed a slight association (Figure S1b,c).

Even though the overall effect size for PFS and NRP1 correlation showed significant heterogeneity between included studies, meta-regression with follow-up as moderator resolved 14.88% of heterogeneity and achieved an assumable residual heterogeneity (I^2^ = 45.56%, Q-test *p =* 0.15). In this case, positive results were not observed with sample size and NOS score as moderators (Table 2) (Figure S1d).

Finally, meta-regression was also performed to assess the heterogeneity sources related to the role of NRP1 overexpression in tumor pathogenesis. However, any of the moderators employed for the analysis could explain or reduce the initially observed heterogeneity (sample size, R^2^=0.00%, I^2^ = 94.94%, Q-test *p <* 0.001; NOS, R^2^ = 0.00%, I^2^ = 91.57%, Q-test *p <* 0.001) (Table 2) (Figure S1e).

### 3.7. Subgroup Analysis

For further analysis to identify the potential sources of heterogeneity, subgroup analysis was also performed using tumor type in addition to sample size, follow-up and NOS score as moderators.

Association analysis between OS and high levels of NRP1 was subjected to subgroup analysis, finding that heterogeneity was only solved when NOS punctuation was higher than 6 (I^2^ = 0.00%, Q-test *p =* 0.81) (Table 3A), similar to the results obtained from meta-regression. Even though significant correlation was found in most of the subgroups, heterogeneity was not decreased by any other moderator. For this reason, we decided to perform subgroup analysis separately for CRC and liver cancer studies. Interestingly, heterogeneity associated with OS and NRP1 meta-analysis in CRC patients was substantially reduced for the subgroup involving studies with less than or equal to 200 patients (I^2^ = 48.01%, Q-test *p =* 0.12); however, NRP1 overexpression was not correlated with OS (HR 1.02, 95% CI 0.99–1.06, *p =* 0.21) (Table 3B). As in meta-regression results, sample size seemed to be a useful moderator for removing heterogeneity. Moreover, the removal of two out of the seven CRC studies [22,33] also resolved heterogeneity (I^2^ = 36.07%, Q-test *p =* 0.18), not showing a significant correlation (HR 1.01, 95% CI 1.00–1.02, *p =* 0.10) again (Table 3B). Notwithstanding, when OS and NRP1 association was analyzed within liver cancer studies, different results were obtained after subgroup analysis (Table 3C). In this regard, in line with meta-regression findings, the NOS scale appeared to be the main covariate responsible for heterogeneity, as subgroups in which NOS punctuation was higher than 5 did not show heterogeneity: NOS>5 (I^2^ = 0.00%, Q-test *p =* 0.72) and NOS > 6/NOS = 7 (I^2^ = 0.00%, Q-test *p =* 0.81). In both cases, high expression of NRP1 was found to be significantly correlated to a lower OS (Table 3C). In this meta-analysis, patients subjected to surgery as well as patients in which no intervention was done were included. Even though subgroups based on this parameter could provide a useful analysis for assessing the heterogeneity source, thus separating patients with and without (R0 resected) cancer for OS, this subgroup analysis could not be performed due to missing data about the intervention made to patients in some of the included studies [29,33].

Although PFS did not show a significant correlation with NRP1, this could be due to the substantial heterogeneity observed (I^2^ = 56.07%, Q-test *p =* 0.09) (Figure 3b). After conducting the analysis of subgroups, heterogeneity was successfully resolved when the sample size was ≤100 (I^2^ = 0.00%, Q-test *p =* 0.97) and removing Benson et al. 2016 (tivozanib/mFOLFOX6 group) (I^2^ = 0.00%, Q-test *p =* 0.74), obtaining only a significant correlation between lower PFS and high NRP1 expression when Benson et al. 2016 (tivozanib/mFOLFOX6 group) was eliminated (Table 3D).

Additionally, subgroup analysis was also carried out to solve heterogeneity in the meta-analysis of NRP1 overexpression and tumor pathogenesis (Table 3E). In this regard, a marked reduction of heterogeneity was achieved when the sample size was greater than 100 patients (I^2^ = 49.09%, Q-test *p =* 0.14) and was solved entirely when selecting studies with NOS score 7 (I^2^ = 0.00%, Q-test *p =* 0.58). Heterogeneity removal led to a significant association of high NRP1 expression with tumor pathogenesis in these cases (Table 3E).

Overall, subgroup analysis led to the resolution of reported heterogeneity for OS, either for all included studies and differentially for CRC and liver cancer studies, for PFS and for tumor pathogenesis. Moreover, heterogeneity removal showed a significant correlation of NRP1 overexpression not only with OS, but also with PFS and tumor pathogenesis.

### 3.8. Analysis of Publication Bias

**Figure 5:**
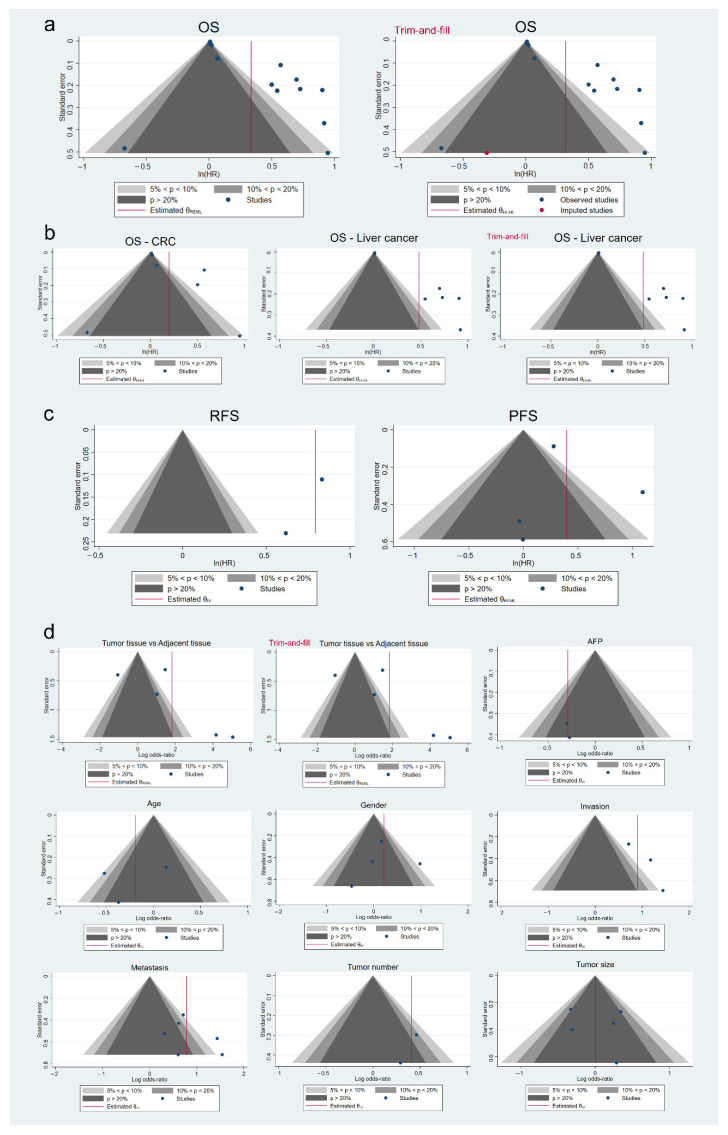
Publication bias evaluation of the correlation of NRP1 overexpression with (**a**) OS for all included studies, (**b**) OS separately for CRC and liver cancer studies, (**c**) RFS and PFS, and (**d**) tumor pathogenesis and the assessed clinicopathological features by funnel plot asymmetry with contour-enhanced funnel plots. Trim-and-fill funnel plots are also included for (**a**) general OS, (**b**) OS in liver cancer studies and (**d**) tumor pathogenesis. AFP, alpha-fetoprotein; CI, confidence interval; CRC, colorectal cancer; HR, hazard ratio; IV, Inverse Variance; OS, overall survival; PFS, progression-free survival; REML, Restricted Maximum Likelihood; RFS, recurrence-free survival.

The risk of bias is a common problem found in meta-analyses due to differences between significant and non-significant published results. After analyzing funnel plot asymmetry together with Egger’s test results, we identified the presence of publication bias in the OS meta-analysis (Table 4) (Figure 5a). Curiously, when CRC and liver cancer studies were separately evaluated, only liver cancer studies showed a significant publication bias (Table 4) (Figure 5b). In both cases, a trim-and-fill method was conducted, which imputed one missing study in global OS and modified global effect size (HR 1.37, 95% CI, 1.13–1.68), while no studies were imputed in OS analysis in liver cancer (Table 4) (Figure 5a-b). Regarding the remaining survival parameters meta-analyzed, statistical analysis did not report the presence of publication bias (Table 4) (Figure 5c).

On the other hand, only studies involved in NRP1 correlation with pathogenesis denoted a substantial risk of bias, not finding significant results among other clinicopathological features analyzed (Table 4) (Figure 5d). After performing sensitivity analysis by trim-and-fill in tumor pathogenesis, any additional study that could be responsible for publication bias was imputed (Table 4) (Figure 5d).

## 4. Discussion

The most frequently diagnosed malignant solid tumors affecting the liver are primary cancers, HCC and CCA, and metastasis from CRC. As a whole, they are second in the rank of the most common and deadly cancers worldwide [3]. Moreover, both liver cancer and CRC are two major gastrointestinal tumors with the highest incidence and mortality rates within these tumor types affecting the digestive system [3]. This was the main reason for performing the present meta-analysis in both tumor types. Despite recent advances in the clinical setting of these tumors, late diagnosis, lack of therapeutic effectiveness and high tumor recurrence rate represent the main factors accounting for their malignancy [4,5,6]. Several investigations have been recently focused on searching and screening for novel biomarkers as an effective diagnostic strategy; however, validation of useful biomarkers in predicting patient prognosis and tumor response remains an urgent need [2,34,35].

In this line of research, elucidating the role of NRP1 in tumor development and progression has been addressed [10,11]. This transmembrane protein is able to modulate cell proliferation, angiogenesis, invasion and cell migration through interaction with multiple growth factors and its receptors, such as VEGF/VEGFR, placenta growth factor (PlGF)/VEGR, hepatocyte growth factor (HGF)/cMET, and transforming growth factor-β1 (TGF-β1)/TGF-βR [10,11,12]. Even though NRP1 is mainly found to be located in the cell membrane inducing cell migration and angiogenesis, soluble isoforms have also been observed. Secreted sNRP1 has demonstrated to modulate the cellular processes of angiogenesis and cell proliferation, exhibiting both promotion and inhibitory effects on tumor angiogenesis and progression, thus increasing the controversy in the role of this protein [11]. Otherwise, NRP1 is broadly expressed in different tissues and increased levels of NRP1 have been observed in numerous tumor types, suggesting a potential role as an oncogenic protein [11]. Therefore, the present meta-analysis aimed at determining the potential role of NRP1 as an independent biomarker in the cancer prognosis of CRC and liver cancer patients, as well as its correlation with several tumor-associated clinicopathological features, in order to evaluate NRP1 as a useful target for the pharmacological landscape of these cancers.

Data from a total of 15 high-quality studies comprising 3407 patients were extracted and included in this meta-analysis, from which 1742 were CRC patients and 1665 suffered from primary liver cancer. Interestingly, as a highly metastatic tumor in the liver [4], from the six CRC studies, three included only metastatic CRC (mCRC) [22,26,28], with one specifically performed with lung and liver mCRC patients [28]. After statistical analysis, pooled results demonstrated that NRP1 overexpression is correlated with a shorter OS in CRC and liver cancer patients. However, liver cancer studies seem to be responsible for this significant association in the general analysis of OS, as mentioned below. When meta-analysis was conducted separately for each tumor type, the statistical significance of this association was only preserved in liver cancer patients but not among CRC studies. Moreover, high levels of NRP1 were also found to be significantly associated with RFS in liver cancer and PFS in CRC patients. The use of meta-analysis has shown to be a valuable tool for assessing the reliability of overexpressed proteins as tumor biomarkers by previous studies performed on CRC and liver cancer [36,37,38]. Although a number of investigations showing this potential correlation in other tumor types have been published [39,40,41,42,43,44,45,46,47,48,49], no previous study has evaluated the role of NRP1 as a prognostic biomarker in CRC and liver cancer patients through meta-analysis. The overexpression of NRP1 in tumor tissue has been correlated with worse prognosis in gastric cancer [39,40], cervical cancer [42], ovarian carcinoma [43], breast cancer [44], non-small cell lung cancer (NSCLC) [45], osteosarcoma [47], bladder cancer [46], glioma [49], nasopharyngeal carcinoma [48], and pancreatic ductal adenocarcinoma [41], among others. Additionally, NRP1 was found to be negatively associated with PFS in gastric cancer [39], NSCLC [45], and nasopharyngeal carcinoma [48], and disease-free survival (DFS) in osteosarcoma [47]. Interestingly, a study performed in patients with HCC who underwent curative hepatic resection analyzed the association of peritumoral expression of NRP1 with OS and time to recurrence (TTR) [50]. That investigation concluded that patients with higher levels of NRP1 in the peritumor tissue, but surprisingly not in the tumor, experienced longer OS and TTR [50]. Along with survival outcomes, NRP1 has been demonstrated to be involved in therapeutic responsiveness in cancer patients [12]. In a study performed with osteosarcoma patients, elevated levels of NRP1 forecasted lower chemotherapeutic response [47], and higher NRP1 plasma levels were established as predictors of bevacizumab efficacy in patients with gastric cancer [51]. Globally, these results support the potential interest of NRP1 as a prognostic biomarker not only in predicting OS but also in other survival-associated parameters and drug response in patients with advanced CRC or liver cancer, which highlights the interesting role of NRP1 as a therapeutic target.

Within the included studies, NRP1 levels determination was performed mainly in the tumor tissue; nevertheless, three articles analyzed serum NRP1 levels [22,26,30]. These results were different but showed interesting findings regarding the potential use of NRP1 as a secreted protein that could be a potential serum biomarker and lead to non-invasive diagnostic procedures. Although results from these articles showed the highest correlations between NRP1 overexpression and OS [22,30] or PFS [26], providing an interesting basis for future studies, further investigations are needed to deeply evaluate the role of NRP1 as a secreted protein and as a potential serum biomarker.

Otherwise, results evaluating the differential expression of NRP1 between tumor and adjacent tissue revealed a significant correlation between NRP1 overexpression and HCC pathogenesis upon analyzing patient subgroups. Similar findings, describing increased NRP1 levels in tumor tissue collected from gastric cancer [40], cervical cancer [42], NSCLC [45], bladder cancer [46], osteosarcoma [47], nasopharyngeal carcinoma [48], and renal cell carcinoma [52] have been reported. Moreover, plasma NRP1 levels have been suggested as a valuable biomarker in breast cancer patients [44]. Altogether, NRP1 seems to be potentially useful in HCC diagnosis and could complement current clinical tools, improving the clinical onset of HCC patients.

Even though no significant association was observed between NRP1 and some clinicopathological features, such as AFP levels, patient age, gender, tumor number and tumor size, NRP1 overexpression was strongly correlated with venous invasion in liver cancer patients, as well as metastasis in CRC and liver cancer patients.

In this regard, only one meta-analysis has been conducted, including gastric cancer studies, in which the NRP1 association with different clinicopathological features was assessed, but prognosis parameters were not analyzed [17]. Similar to our findings, that study observed a significant correlation between high NRP1 levels and III-IV stages of tumor-node-metastasis (TNM) classification, poor differentiation and lymph node metastasis, with an absence of association with tumor size greater than 5 cm [17].

Along these lines, when patients with NSCLC were classified into low and high NRP1 expression groups, a strong correlation was identified between histological grade, TNM stages and lymph node metastasis with high NRP1 levels [45]. However, as observed in the present meta-analysis, no significant correlation between NRP1 expression and patient age, gender and pathology type was found [45]. Additional reports from studies conducted in different tumor types revealed that NRP1 overexpression was directly related to the presence of distant metastasis [40,47,48], advanced stages [47,48], invasion depth and lymph node metastasis in gastric cancer [40], osteosarcoma [47] and nasopharyngeal carcinoma [48], thus supporting results obtained in the present meta-analysis. Moreover, the liver is the main organ affected by CRC metastasis [4], and three out of the six CRC studies included in this meta-analysis were performed with patients suffering from mCRC [22,26,28]. However, in preclinical models, similar results also demonstrated that NRP1 removal led to higher cell proliferation [42] and lower cell migration, invasion [40,42,53], epithelial-to-mesenchymal transition (EMT) [40] and angiogenesis [53] in cervical cancer [42], NSCLC [53] and gastric cancer [40]. Despite further studies needing to be accomplished to deeply elucidate the role of NRP1 in invasive-related mechanisms, this protein seems to have a crucial role in modulating invasive processes in different cancer types, including CRC and liver cancer. This supports the interest of NRP1 as a potential therapeutic target to overcome these tumor-associated characteristics.

The potential use of NRP1 as a prognostic biomarker or therapeutic target leads to controversial interpretations of the suitability of this protein as one of these clinical tools. In the present study, we found that NRP1 was significantly correlated with tumor pathogenesis and also with several clinicopathological features that place it as an interesting biomarker and target, respectively. Several investigations have also described the key role of NRP1 in cell response to chemotherapy, reinforcing the interesting use of NRP1 as a therapeutic target [10,54]. However, a lower number of investigations have determined the role of NRP1 in the process of tumor pathogenesis, describing that NRP1 could drive nutrient supply to tumor cells through angiogenesis induction, therefore leading to cell survival and proliferation, and in consequence to tumor formation [13]. Moreover, a preclinical study employed NRP1 knockdown human hepatoma cells to generate a mouse model of HCC, observing that NRP1 loss reduced the tumor volume when compared to non-transfected hepatoma cells, providing evidence of the role of NRP1 in tumor development and pathogenesis [48]. Nonetheless, in this line few studies have been conducted in order to clarify the most suitable use of NRP1, as either biomarker or therapeutic target, highlighting the necessity of future investigations.

As previously mentioned, the primary NRP1 role in cancer cells is related to its ability to interact with relevant growth factors and the corresponding receptors in tumor cells, thus promoting cell proliferation, migration, angiogenesis and EMT [10]. Among the ligands of NRP1, VEGF-A is the best-known binding partner that mediates the pro-angiogenic and pro-invasive role of NRP1 in cancer through a co-interaction with NRP1/VEGFR2 [11]. Together with the association found between high levels of NRP1 and invasion and metastasis, two of the included studies reported that NRP1 expression is positively correlated with VEGFR2 in HCC [27], and that not only NRP1, but also VEGF and VEGFR1/3 overexpression, were associated with poor prognosis in CRC patients [22]. Moreover, tivozanib, a selective inhibitor of the VEGFR1-3 isoforms, showed to be as effective as bevacizumab in combination with mFOLFOX6 treatment against untreated CRC [26]. In that study, longer OS was achieved when NRP1 levels were classified as low in the tivozanib/mFOLFOX6 group, which highlights the synergistic effect of inhibited expression of VEGFR and lower levels of NRP1 [26].

Regarding previously published results, a study performed with HCC patients evaluating the peritumoral expression of NRP1 reported that, as observed with high NRP1 levels, VEGFR2 overexpression in peritumoral tissue was associated with lower recurrence probability and higher OS [50]. NSCLC patients in which NRP1 was overexpressed also showed increased levels of VEGFR2 and a significant correlation between both proteins [45]. Furthermore, preclinical studies support these findings. The blockade of NRP1-VEGFR2 interaction led to restriction of tumor growth and angiogenesis in a mouse model of CRC [55], while targeting NRP1 with a specific antagonist prevented migration induction in CRC cells adapted to the treatment with the VEGFR-targeting drug sunitinib [54]. Therefore, NRP1 may act as a relevant protein in the modulation of invasion and related processes, being highly associated with the well-known VEGF/VEGFR2 signaling pathway.

The present study represents the first meta-analysis in which the potential role of NRP1 in CRC and liver cancer prognosis has been evaluated. Moreover, there have also been no previous articles that meta-analyzed NRP1 as a useful diagnostic biomarker for these tumors. As part of the research, further analysis to assess the presence of heterogeneity and publication bias was performed, and sources of heterogeneity were widely addressed through two complementary methods, subgroup analysis and meta-regression. Nonetheless, our study has some limitations that need to be described to improve future investigation in this field. As exclusion criteria, no English full-text availability led to the discard of two articles written in Chinese, and this could account for publication bias as essential data for this meta-analysis might have been missed. Due to the relevance of etiology in both tumor types, CRC and liver cancer, the absence of complete information about the patient’s etiology in the included studies also limits the interpretation of the results obtained. Primary tumor site was fully described in the included studies; nevertheless, among the CRC studies, three included only patients with mCRC and the other three did not discriminate between metastatic and non-metastatic tumor, increasing the variability of the study results. Moreover, the measurement method of NRP1 levels supposes a critical limitation, since studies that classified patients regarding both mRNA and protein levels of NRP1 were included for assessment. Along with this, the correlation of prognosis and other tumor-related parameters was evaluated with high NRP1 levels in either tissue or serum samples, increasing the uncertainty of the results. Globally, a suitable number of studies was obtained; nonetheless, for each tumor type a lower number was included for the meta-regression and publication bias assessment. These types of analysis provide more accurate results with pooled data from ten or more articles, therefore it could be a limitation of the analysis performed. Although several articles used similar criteria for the definition of high and low NRP1 groups, differences in this regard could also account for the variability in the interpretation of obtained results. Some articles directly reported data that were used for meta-analyzing; however, estimated values from Kaplan–Meier curves of some studies were also included, contributing to an increased deviation of estimated results from real overall effect size. Furthermore, all included studies did not provide data for all analyzed parameters, and some prognostic analyses, such as RFS and PFS, were included in a low number of studies, which could lead to inaccurate results.

## 5. Conclusions

In summary, this systematic review with meta-analysis summarized and evaluated the potential correlation of high NRP1 expression with worse prognosis and tumor-associated clinicopathological characteristics in CRC and liver cancer patients. Approximately half (50.40%) of the patients included in this study showed NRP1 overexpression, showing a significant association between NRP1 high levels and both enhanced tumor malignant characteristics and consistent shorter survival. Overall, these results support the growing interest in NRP1 as a useful diagnostic and prognostic biomarker, as well as its potential as a pharmacological target, in both CRC and primary liver tumors.

## Figures and Tables

**Table 2:** Assessment of heterogeneity by meta-regression in global OS, CRC and liver cancer OS, PFS and tumor pathogenesis.

Variable	Beta Coefficient	z	*p*-Value	95% CI	Residual Heterogeneity		
					I^2^	Q Test *p*-Value	R^2^
OS							
Sample size	1.00	0.66	0.51	0.999–1.001	99.68%	0.00	0.00%
Follow-up	1.00	−0.96	0.34	0.992–1.003	99.74%	0.00	2.26%
NOS	1.28	2.32	0.02	1.038–1.570	99.68%	0.00	42.46%
OS, CRC							
Sample size	1.00	2.17	0.03	1.000–1.002	92.07%	0.00	59.33%
Follow-up	1.00	−0.60	0.55	0.993–1.004	98.69%	0.00	0.00%
NOS	1.03	0.11	0.91	0.591–1.802	99.08%	0.00	0.00%
OS, Liver cancer							
Sample size	1.00	−0.20	0.84	0.997–1.003	85.16%	0.00	0.00%
Follow-up	1.00	−0.27	0.78	0.987–1.010	86.76%	0.00	0.00%
NOS	1.32	2.36	0.02	1.048–1.665	99.61%	0.03	61.08%
PFS							
Sample size	1.00	−0.14	0.89	0.997–1.002	59.73%	0.07	0.00%
Follow-up	0.98	−0.94	0.35	0.941–1.022	45.56%	0.15	14.88%
NOS	0.63	−0.59	0.55	0.135–2.921	68.88%	0.05	0.00%
Tumor tissue vs. Adjacent tissue							
Sample size	1.00	0.45	0.65	0.983–1.028	94.94%	0.00	0.00%
NOS	0.32	−0.46	0.64	0.002–41.134	91.57%	0.00	0.00%

CI, confidence interval; CRC, colorectal cancer; NOS, Newcastle-Ottawa scale; OS, overall survival; PFS, progression-free survival.

**Table 3:** Subgroup analysis of prognostic and tumor pathogenesis correlation with NRP1 overexpression.

**A.** ** OS**									
**Subgroup**	**Studies (n)**	**Cases (n)**		**Pooled HR**			**Test for Heterogeneity**		**Model Used**
				**HR**	**95% CI**	***p*- ** **Value**	**I^2^**	**Q Test *p*-Value**	
Tumor type									
CRC	7	1537		1.22	0.96–1.54	0.11	98.52%	0.00	REM
Liver cancer	7	1278		1.62	1.18–2.21	0.00 *	99.82%	0.00	REM
Tumor type									
CCA	2	327		1.39	0.71–2.74	0.34	93.68%	0.00	REM
CRC	7	1537		1.22	0.96–1.54	0.11	98.52%	0.00	REM
HCC	5	951		1.75	1.20–2.56	0.00 *	82.97%	0.00	REM
Sample size									
≤100	4	257		1.01	0.99–1.02	0.41	0.01%	0.03	REM
>100	10	2558		1.52	1.21–1.91	0.00 *	99.78%	0.00	REM
≤200	8	779		1.36	0.98–1.88	0.06	99.14%	0.00	REM
>200	6	2036		1.46	1.11–1.93	0.01 *	99.85%	0.00	REM
≤300	12	1867		1.42	1.13–1.80	0.00 *	99.58%	0.00	REM
>300	2	948		1.32	0.76–2.29	0.32	96.26%	0.00	REM
≤400	13	2232		1.37	1.11–1.70	0.00 *	99.82%	0.00	REM
>400	1	583		1.77	1.43–2.19	—	—	—	—
NOS scale									
5	6	1521		1.17	0.94–1.45	0.15	99.86%	0.00	REM
6	5	659		1.45	0.90–2.36	0.13	83.08%	0.00	REM
7	3	635		1.95	1.55–2.44	0.00 *	0.00%	0.81	FEM ^†^
NOS scale (threshold 5)									
≤5	6	1521		1.17	0.94–1.45	0.15	99.86%	0.00	REM
>5	8	1294		1.64	1.25–2.16	0.00 *	72.52%	0.00	REM
NOS scale (threshold 6)									
≤6	11	2180		1.28	1.03–1.60	0.03 *	99.83%	0.00	REM
>6	3	635		1.95	1.55–2.44	0.00 *	0.00%	0.81	FEM ^†^
Follow up (months)									
≤60	3	806		1.62	1.01–2.60	0.04 *	91.42%	0.00	REM
>60	11	2009		1.34	1.06–1.68	0.01 *	99.84%	0.00	REM
≤120	8	1842		1.49	1.15–1.92	0.00 *	99.75%	0.00	REM
>120	6	973		1.27	0.89–1.81	0.19	99.26%	0.00	REM
**B. OS in CRC studies**									
**Subgroup**	**Studies (n)**	**Cases (n)**		**Pooled HR**			**Test for heterogeneity**		**Model used**
				**HR**	**95% CI**	***p*- ** **value**	**I^2^**	**Q test *p*-value**	
Sample size									
≤100	2	128		0.86	0.44–1.67	0.66	56.20%	0.13	REM
>100	5	1409		1.34	1.00–1.81	0.054	99.15%	0.00	REM
≤200	4	396		1.02	0.99–1.06	0.21	48.01%	0.12	FEM ^†^
>200	3	1141		1.40	0.96–2.04	0.08	91.32%	0.00	REM
≤300	6	954		1.01	1.00–1.02	0.09	0.00%	0.03	REM
>300	1	583		1.77	1.43–2.19	—	—	—	—
NOS scale									
5	3	1027		1.20	0.85–1.71	0.30	99.58%	0.00	REM
6	4	510		1.24	0.77–2.00	0.37	74.05%	0.02	REM
Follow up (months)									
≤120	2	657		1.37	0.84–2.24	0.21	92.86%	0.00	REM
>120	5	880		1.13	0.87–1.47	0.37	98.57%	0.02	REM
Without Spencer et al. 2013 and Liu et al. 2021									
	5	675		1.01	1.00–1.02	0.10	36.07%	0.18	FEM ^†^
**C. OS in liver cancer studies**									
**Subgroup**	**Studies (n)**	**Cases (n)**		**Pooled HR**			**Test for heterogeneity**		**Model used**
				**HR**	**95% CI**	***p*- ** **value**	**I^2^**	**Q test *p*-value**	
Sample size									
≤100	2	129		1.47	0.61–3.56	0.39	83.51%	0.01	REM
>100	5	1149		1.71	1.21–2.42	0.00 *	85.26%	0.00	REM
≤200	4	383		1.70	1.07–2.71	0.03 *	84.83%	0.00	REM
>200	3	895		1.56	0.96–2.54	0.08	89.89%	0.00	REM
≤300	6	913		1.78	1.29–2.45	0.00 *	82.71%	0.00	REM
>300	1	365		1.01	1.00–1.02	—	—	—	—
NOS scale									
5	3	494		1.18	0.80–1.74	0.41	99.91%	0.05	REM
6	1	149		2.47	1.60–3.81	—	—	—	—
7	3	635		1.95	1.55–2.44	0.00 *	0.00%	0.81	FEM ^†^
NOS scale (threshold 5)									
≤5	3	494		1.18	0.80–1.74	0.41	99.91%	0.05	REM
>5	4	784		2.05	1.67–2.50	0.00 *	0.00%	0.72	FEM ^†^
NOS scale (threshold 6)									
≤6	4	643		1.47	0.89–2.43	0.13	99.94%	0.00	REM
>6	3	635		1.95	1.55–2.44	0.00 *	0.00%	0.81	FEM ^†^
Follow up (months)									
≤60	1	149		2.47	1.60–3.81	—	—	—	—
>60	6	1129		1.51	1.09–2.08	0.01 *	99.83%	0.00	REM
≤120	6	1185		1.55	1.12–2.15	0.01 *	99.85%	0.00	REM
>120	1	93		2.51	1.21–5.18	—	—	—	—
**D. PFS**									
**Subgroup**	**Studies (n)**	**Cases (n)**		**Pooled HR**			**Test for heterogeneity**		**Model used**
				**HR**	**95% CI**	***p*- ** **value**	**I^2^**	**Q test *p*-value**	
Sample size									
≤100	2	128		0.98	0.47–2.04	0.95	0.00%	0.97	FEM ^†^
>100	2	691		1.86	0.85–4.10	0.12	82.09%	0.02	REM
≤200	3	236		1.56	0.70–3.49	0.28	58.06%	0.09	REM
>200	1	583		1.32	1.11–1.57	—	—	—	—
NOS scale									
5	3	745		1.59	0.88–2.86	0.12	68.88%	0.05	REM
6	1	74		1	0.32–3.14	—	—	—	—
Without Benson et al. 2016 (Tivozanib/mFOLFOX6 group)									
	3	711		1.3	1.10–1.54	0.00 *	0.00%	0.74	FEM ^†^
**E. Tumor tissue *vs.* Adjacent tissue**									
**Subgroup**	**Studies (n)**	**Cases (n)**	**Cases with high NRP1 expression (%)**	**Pooled OR**			**Test for heterogeneity**		**Model used**
				**OR**	**95% CI**	***p*- ** **value**	**I^2^**	**Q test *p*-value**	
Sample size									
≤100	2	94	88.30	6.25	0.02–2479.82	0.55	93.81%	0.00	REM
>100	3	652	38.34	4.48	2.59–7.75	0.00 *	49.09%	0.14	FEM ^†^
≤200	3	199	80.90	4.87	0.18–130.34	0.34	97.06%	0.00	REM
>200	2	547	31.44	10.54	0.50–222.18	0.13	73.98%	0.05	REM
≤300	4	438	40.41	3.85	0.42–35.34	0.23	94.33%	0.00	REM
>300	1	308	50.65	64.65	3.92–1065.96	—	—	—	—
NOS scale									
6	3	402	59.45	12.25	0.24–619.71	0.21	90.15%	0.00	REM
7	2	344	27.33	4.03	2.31–7.05	0.00 *	0.00%	0.58	FEM ^†^

CCA, cholangiocarcinoma; CI, confidence Interval; CRC, colorectal cancer; FEM, fixed-effects model; HCC, hepatocellular carcinoma; HR, hazard ratio; NOS, Newcastle-Ottawa scale; OR, odds ratio; OS, overall survival; PFS, progression-free survival; REM, random-effects model. * Significant correlation, *p*-value < 0.05. ^†^ High heterogeneity solved (I^2^ < 50% and Q test *p*-value > 0.10).

**Table 4:** Evaluation of risk of publication bias.

**Survival Parameter**	**Studies (n)**	**Egger’s Test (*p*-Value)**	**Model Used**	**Trim-and-Fill Analysis**		**Studies Imputed (n)**
				**HR**	**95% CI**	
OS	14	0.00 *	REM	1.37	1.13–1.68	1
PFS	4	0.74	REM	—	—	—
RFS	2	0.40	FEM	—	—	—
OS for CRC	7	0.57	REM	—	—	—
OS for liver cancer	7	0.00*	REM	1.62	1.18–2.21	0
**Clinicopathological feature**	**Studies (n)**	**Egger’s test (*p*-value)**	**Model used**	**Trim-and-fill analysis**		**Studies imputed (n)**
				**HR**	**95% CI**	
Tumor tissue vs. Adjacent tissue	5	0.01 *	REM	6.19	0.77–49.59	0
AFP	2	0.96	FEM	—	—	—
Age	3	0.38	FEM	—	—	—
Gender	4	0.87	FEM	—	—	—
Invasion	3	0.24	FEM	—	—	—
Metastasis	6	0.44	FEM	—	—	—
Tumor number	2	0.75	FEM	—	—	—
Tumor size	5	0.68	FEM	—	—	—

AFP, alpha-fetoprotein; CI, confidence interval; CRC, colorectal cancer; FEM, fixed-effects model; HR, hazard ratio; OR, odds ratio; OS, overall survival; PFS, progression-free survival; REM, random-effects model; RFS, recurrence-free survival. * Significant publication bias, *p*-value < 0.05.

## Supplementary Materials

The following supporting information can be downloaded at: https://www.mdpi.com/article/10.3390/cancers14143455/s1, Table S1: PRISMA 2020 checklist, Table S2: PRISMA 2020 for Abstracts checklist, Table S3: Full search strategy employed for each database (up to and including 31 May 2022), Table S4: Staining procedure and antibodies used in the included studies analyzing NRP1 expression by IHC, Figure S1: Bubbleplots for the representation of the meta-regression performed for (**a**) global OS, (**b**) OS in CRC studies, (**c**) OS in liver cancer studies, (**d**) PFS and (**e**) tumor pathogenesis.
