# Supplementary material for: Neuropilin-1 as a Potential Biomarker of Prognosis and Invasive-Related Parameters in Liver and Colorectal Cancer: A Systematic Review and Meta-Analysis of Human Studies
Source: Cancers (Basel). 2022 Jul 15;14(14):3455. doi: 10.3390/cancers14143455 (PMC9318974; doi:10.3390/cancers14143455)
Supplement: Supplementary file 1 [file cancers-14-03455-s001.zip › cancers-1793764-supplementary.pdf]

Table S1. PRISMA 2020 checklist.

Section and Topic	Item #	Checklist Item	Location Where Item is Reported
**TITLE**			
Title	1	Identify the report as a systematic review.	Page 1
**ABSTRACT**			
Abstract	2	See the PRISMA 2020 for Abstracts checklist.	Page 1 and Table S2
**INTRODUCTION**			
Rationale	3	Describe the rationale for the review in the context of existing knowledge.	Page 2
Objectives	4	Provide an explicit statement of the objective(s) or question(s) the review addresses.	Page 2
**METHODS**			
Eligibility criteria	5	Specify the inclusion and exclusion criteria for the review and how studies were grouped for the syntheses.	Page 3
Information sources	6	Specify all databases, registers, websites, organisations, reference lists and other sources searched or consulted to identify studies. Specify the date when each source was last searched or consulted.	Page 3
Search strategy	7	Present the full search strategies for all databases, registers and websites, including any filters and limits used.	Page 3 and Table S3
Selection process	8	Specify the methods used to decide whether a study met the inclusion criteria of the review, including how many reviewers screened each record and each report retrieved, whether they worked independently, and if applicable, details of automation tools used in the process.	Page 3
Data collection process	9	Specify the methods used to collect data from reports, including how many reviewers collected data from each report, whether they worked independently, any processes for obtaining or confirming data from study investigators, and if applicable, details of automation tools used in the process.	Page 3

Section and Topic	Item #	Checklist Item	Location Where Item is Reported
Data items	10a	List and define all outcomes for which data were sought. Specify whether all results that were compatible with each outcome domain in each study were sought (e.g. for all measures, time points, analyses), and if not, the methods used to decide which results to collect.	Pages 3-4
	10b	List and define all other variables for which data were sought (e.g. participant and intervention characteristics, funding sources). Describe any assumptions made about any missing or unclear information.	Pages 3-4 and Table S4
Study risk of bias assessment	11	Specify the methods used to assess risk of bias in the included studies, including details of the tool(s) used, how many reviewers assessed each study and whether they worked independently, and if applicable, details of automation tools used in the process.	Page 3
Effect measures	12	Specify for each outcome the effect measure(s) (e.g. risk ratio, mean difference) used in the synthesis or presentation of results.	Page 3
Synthesis methods	13a	Describe the processes used to decide which studies were eligible for each synthesis (e.g. tabulating the study intervention characteristics and comparing against the planned groups for each synthesis (item #5)).	Pages 3-4
	13b	Describe any methods required to prepare the data for presentation or synthesis, such as handling of missing summary statistics, or data conversions.	Pages 3-4
	13c	Describe any methods used to tabulate or visually display results of individual studies and syntheses.	Pages 3-4
	13d	Describe any methods used to synthesize results and provide a rationale for the choice(s). If meta-analysis was performed, describe the model(s), method(s) to identify the presence and extent of statistical heterogeneity, and software package(s) used.	Pages 3-4
	13e	Describe any methods used to explore possible causes of heterogeneity among study results (e.g. subgroup analysis, meta-regression).	Pages 3-4
	13f	Describe any sensitivity analyses conducted to assess robustness of the synthesized results.	Pages 3-4
Reporting bias assessment	14	Describe any methods used to assess risk of bias due to missing results in a synthesis (arising from reporting biases).	Page 4
Certainty assessment	15	Describe any methods used to assess certainty (or confidence) in the body of evidence for an outcome.	-

Section and Topic	Item #	Checklist Item	Location Where Item is Reported
**RESULTS**			
Study selection	16a	Describe the results of the search and selection process, from the number of records identified in the search to the number of studies included in the review, ideally using a flow diagram.	Page 4 and Figure 1
	16b	Cite studies that might appear to meet the inclusion criteria, but which were excluded, and explain why they were excluded.	Page 4 and Figure 1
Study characteristics	17	Cite each included study and present its characteristics.	Page 4, and Table 1
Risk of bias in studies	18	Present assessments of risk of bias for each included study.	Page 4 and Table 1
Results of individual studies	19	For all outcomes, present, for each study: (a) summary statistics for each group (where appropriate) and (b) an effect estimate and its precision (e.g. confidence/credible interval), ideally using structured tables or plots.	Figures 2, 3 and 4
Results of syntheses	20a	For each synthesis, briefly summarise the characteristics and risk of bias among contributing studies.	Table 3 and Figures 2, 3 and 4
	20b	Present results of all statistical syntheses conducted. If meta-analysis was done, present for each the summary estimate and its precision (e.g. confidence/credible interval) and measures of statistical heterogeneity. If comparing groups, describe the direction of the effect.	Pages 8-17, Tables 2 and 3, and Figures 2, 3 and 4
	20c	Present results of all investigations of possible causes of heterogeneity among study results.	Pages 10-17 Tables 2 and 3, and Fig-

Section and Topic	Item #	Checklist Item	Location Where Item is Reported
			ure S1
	20d	Present results of all sensitivity analyses conducted to assess the robustness of the synthesized results.	Page 18 and Table 4
Reporting biases	21	Present assessments of risk of bias due to missing results (arising from reporting biases) for each synthesis assessed.	Page 18, Table 4 and Figure 5
Certainty of evidence	22	Present assessments of certainty (or confidence) in the body of evidence for each outcome assessed.	-
**DISCUSSION**			
Discussion	23a	Provide a general interpretation of the results in the context of other evidence.	Pages 20-22
	23b	Discuss any limitations of the evidence included in the review.	Page 23
	23c	Discuss any limitations of the review processes used.	Page 23
	23d	Discuss implications of the results for practice, policy, and future research.	Pages 20-22
**OTHER INFORMATION**			
Registration and protocol	24a	Provide registration information for the review, including register name and registration number, or state that the review was not registered.	Pages 1-3
	24b	Indicate where the review protocol can be accessed, or state that a protocol was not prepared.	Pages 1-3
	24c	Describe and explain any amendments to information provided at registration or in the protocol.	-
Support	25	Describe sources of financial or non-financial support for the review, and the role of the funders or sponsors in the review.	Page 24
Competing interests	26	Declare any competing interests of review authors.	Page 24

Section and Topic	Item #	Checklist Item	Location Where Item is Reported
Availability of data, code and other materials	27	Report which of the following are publicly available and where they can be found: template data collection forms; data extracted from included studies; data used for all analyses; analytic code; any other materials used in the review.	-

Table S2. PRISMA 2020 for Abstracts checklist.

Section and Topic	Item #	Checklist Item	Reported (Yes/No)
**TITLE**			
Title	1	Identify the report as a systematic review.	-
**BACKGROUND**			
Objectives	2	Provide an explicit statement of the main objective(s) or question(s) the review addresses.	Yes
**METHODS**			
Eligibility criteria	3	Specify the inclusion and exclusion criteria for the review.	No
Information sources	4	Specify the information sources (e.g. databases, registers) used to identify studies and the date when each was last searched.	Yes
Risk of bias	5	Specify the methods used to assess risk of bias in the included studies.	No
Synthesis of results	6	Specify the methods used to present and synthesise results.	Yes
**RESULTS**			
Included studies	7	Give the total number of included studies and participants and summarise relevant characteristics of studies.	No
Synthesis of results	8	Present results for main outcomes, preferably indicating the number of included studies and participants for each. If meta-analysis was done, report the summary estimate and confidence/credible interval. If comparing groups, indicate the direction of the effect (i.e. which group is favoured).	Yes

Section and Topic	Item #	Checklist Item	Reported (Yes/No)
**DISCUSSION**			
Limitations of evidence	9	Provide a brief summary of the limitations of the evidence included in the review (e.g. study risk of bias, inconsistency and imprecision).	No
Interpretation	10	Provide a general interpretation of the results and important implications.	Yes
**OTHER**			
Funding	11	Specify the primary source of funding for the review.	No
Registration	12	Provide the register name and registration number.	Yes

Table S3. Full search strategy employed for each database (up to and including May 31st, 2022).

DATABASE	SEARCH STRATEGY
**PubMed**	("nrp1"[All Fields] OR "nrp-1"[All Fields] OR "nrp 1"[All Fields] OR "neuropilin 1"[All Fields] OR "neuropilin-1"[All Fields] OR "CD304"[All Fields] OR "VEGF165R"[All Fields]) AND ("hepatocellular carcinoma"[All Fields] OR "hepatocarcinoma"[All Fields] OR "HCC"[All Fields] OR "liver tumor"[All Fields] OR "hepatic tumor"[All Fields] OR "liver cancer"[All Fields] OR "hepatic cancer"[All Fields] OR "cholangiocarcinoma"[All Fields] OR "CCA"[All Fields] OR "hepatoma"[All Fields] OR "hepatoblastoma"[All Fields] OR "angiosarcoma"[All Fields] OR "colorectal cancer"[All Fields] OR "colon cancer"[All Fields] OR "CRC"[All Fields] OR "mCRC"[All Fields] OR "colon adenocarcinoma"[All Fields])
**Scopus**	TITLE-ABS-KEY (("nrp1" OR "nrp-1" OR "nrp 1" OR "neuropilin 1" OR "neuropilin-1" OR "CD304" OR "VEGF165R") AND ("hepatocellular carcinoma" OR "hepatocarcinoma" OR "HCC" OR "liver tumor" OR "hepatic tumor" OR "liver cancer" OR "hepatic cancer" OR "cholangiocarcinoma" OR "CCA" OR "hepatoma" OR "hepatoblastoma" OR "angiosarcoma" OR "colorectal cancer" OR "colon cancer" OR "CRC" OR "mCRC" OR "colon adenocarcinoma"))
**Web of Science Core Collection**	TS=(("nrp1" OR "nrp-1" OR "nrp 1" OR "neuropilin 1" OR "neuropilin-1" OR "CD304" OR "VEGF165R") AND ("hepatocellular carcinoma" OR "hepatocarcinoma" OR "HCC" OR "liver tumor" OR "hepatic tumor" OR "liver cancer" OR "hepatic cancer" OR "cholangiocarcinoma" OR "CCA" OR "hepatoma" OR "hepatoblastoma" OR "angiosarcoma" OR "colorectal cancer" OR "colon cancer" OR "CRC" OR "mCRC" OR "colon adenocarcinoma")) *Indexes=SCI-EXPANDED, SSCI, A&HCI, CPCI-S, CPCI-SSH, BKCI-S, BKCI-SSH, ESCI, CCR-EXPANDED, IC* *Timespan=All years*
**Excerpta Medica**	('nrp1' OR 'nrp-1' OR 'nrp 1' OR 'neuropilin 1'/exp OR 'neuropilin 1' OR 'neuropilin-1' OR 'neuropilin-1' OR 'cd304' OR 'vegf165r') AND ('hepatocellular carcinoma' OR

**Database**	'hepatocellular carcinoma' OR 'hepatocarcinoma' OR 'hepatocarcinoma' OR 'hcc' OR 'liver tumor' OR 'liver tumor' OR 'hepatic tumor' OR 'hepatic tumor' OR 'liver cancer' OR 'liver cancer' OR 'hepatic cancer' OR 'hepatic cancer' OR 'cholangiocarcinoma' OR 'cholangiocarcinoma' OR 'cca' OR 'hepatoma' OR 'hepatoma' OR 'hepatoblastoma' OR 'hepatoblastoma' OR 'angiosarcoma' OR 'angiosarcoma' OR 'colorectal cancer' OR 'colorectal cancer' OR 'colon cancer' OR 'colon cancer' OR 'crc' OR 'mcrc' OR 'colon adenocarcinoma' OR 'colon adenocarcinoma')
**Cochrane Library**	("nrp1" OR "nrp-1" OR "nrp 1" OR "neuropilin 1" OR "neuropilin-1" OR "CD304" OR "VEGF165R") AND ("hepatocellular carcinoma" OR "hepatocarcinoma" OR "HCC" OR "liver tumor" OR "hepatic tumor" OR "liver cancer" OR "hepatic cancer" OR "cholangiocarcinoma" OR "CCA" OR "hepatoma" OR "hepatoblastoma" OR "angiosarcoma" OR "colorectal cancer" OR "colon cancer" OR "CRC" OR "mCRC" OR "colon adenocarcinoma"):ti, ab, kw

Table S4. Antibodies and procedure of staining employed by those included articles that analyzed NRP1 expression using IHC.

Study	Publication Year	Tumor Type	Antibody	Dilution	Source	Reference
Li et al. [32]	2021	HCC	Rabbit monoclonal	1:100	Abcam	NR
Bianconi et al. [28]	2020	CRC	Rabbit monoclonal (EPR3113)	1:200	Abcam	ab81321
Wu et al. [23]	2020	CCA	Rabbit monoclonal	1:200	Abcam	NR
Zhu et al. [16]	2020	CCA	Rabbit anti-human	1:250	Santa Cruz Biotechnology Inc.	sc-5307
Lin et al. [14]	2018	HCC	Rabbit monoclonal	NR	Abcam	ab81321
Zhang et al. [25]	2016	HCC	Rabbit monoclonal (A6)	1:100	Self produced	NR
Berge et al. [27]	2011	HCC	Rabbit polyclonal	1:50	Invitrogen (Zymed laboratories)	34-7300
Ochiumi et al. [21]	2006	CRC	Mouse monoclonal A-12	1:100	Santa Cruz Biotechnology Inc.	NR

CCA, cholangiocarcinoma; CRC, colorectal cancer; HCC, hepatocellular carcinoma; NR, not reported.

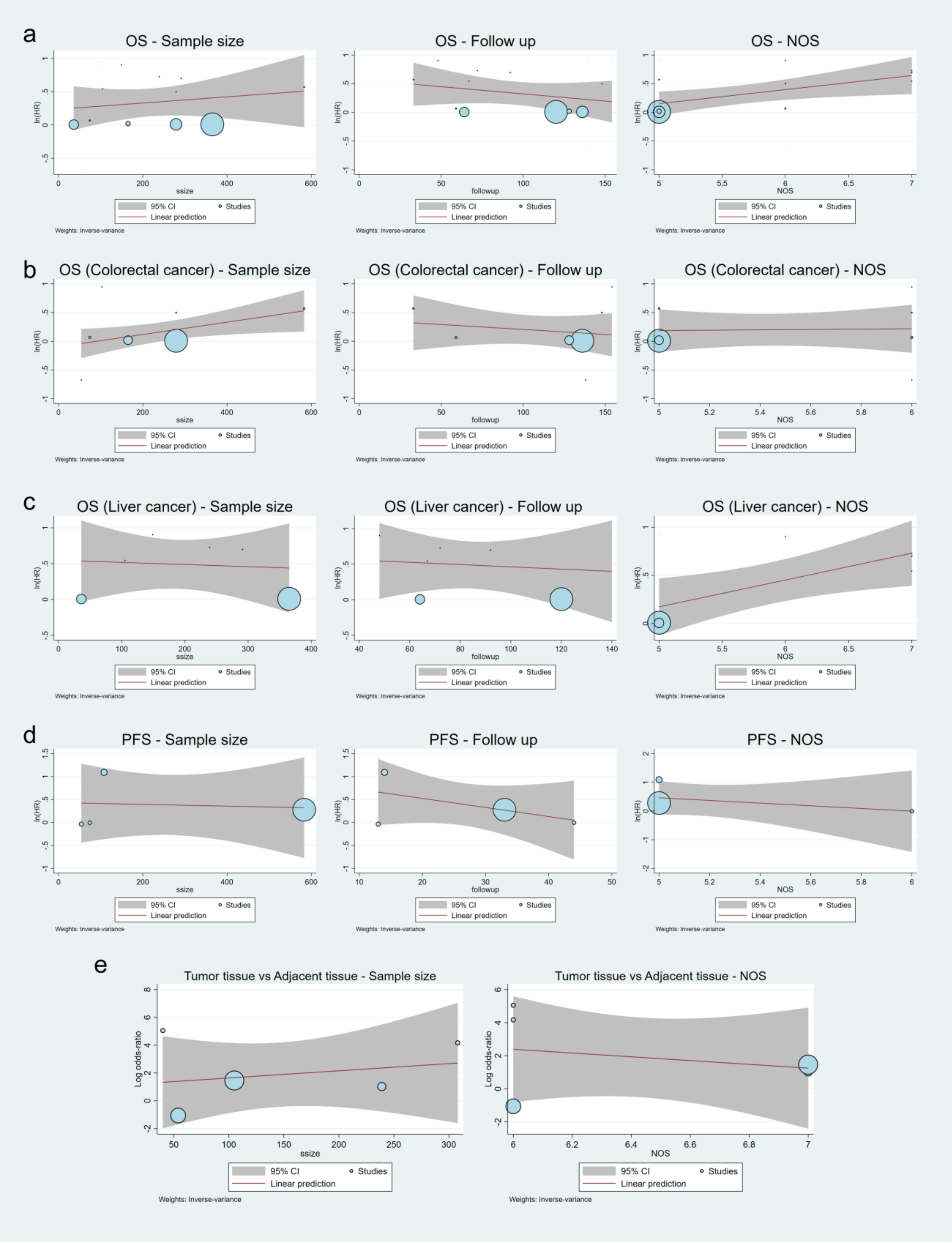

Figure S1. Bubbleplots for the representation of the meta-regression performed for (**a**) global OS, (**b**) OS in CRC studies, (**c**) OS in liver cancer studies, (**d**) PFS and (**e**) tumor pathogenesis. For (**a**)–(**d**), sample size, follow up and NOS score were used as moderators; for (**e**), sample size and NOS score were used as moderators. CI, confidence interval; CRC, colorectal cancer; NOS, Newcastle-Ottawa score; OS, overall survival; PFS, progression-free survival.
